# Innate Functions of Immunoglobulin M Lessen Liver Gene Transfer with Helper-Dependent Adenovirus

**DOI:** 10.1371/journal.pone.0085432

**Published:** 2014-01-21

**Authors:** Carmen Unzu, Ignacio Melero, Aizea Morales-Kastresana, Ana Sampedro, Irantzu Serrano-Mendioroz, Arantza Azpilikueta, María Carmen Ochoa, Juan Dubrot, Eduardo Martínez-Ansó, Antonio Fontanellas

**Affiliations:** 1 Gene Therapy and Hepatology Area, Centre for Applied Medical Research (CIMA), University of Navarra, Pamplona, Spain; 2 Department of Pathology and Immunology, University of Geneva Medical School, Geneva, Switzerland; Mayo Clinic, United States of America

## Abstract

The immune system poses obstacles to viral vectors, even in the first administration to preimmunized hosts. We have observed that the livers of B cell-deficient mice were more effectively transduced by a helper-dependent adenovirus serotype-5 (HDA) vector than those of WT mice. This effect was T-cell independent as shown in athymic mice. Passive transfer of the serum from adenovirus-naïve WT to Rag1KO mice resulted in a reduction in gene transfer that was traced to IgM purified from serum of adenovirus-naïve mice. To ascribe the gene transfer inhibition activity to either adenoviral antigen-specific or antigen-unspecific functions of IgM, we used a monoclonal IgM antibody of unrelated specificity. Both the polyclonal and the irrelevant monoclonal IgM inhibited gene transfer by the HDA vector to either cultured hepatocellular carcinoma cells or to the liver of mice *in vivo*. Adsorption of polyclonal or monoclonal IgMs to viral capsids was revealed by ELISAs on adenovirus-coated plates. These observations indicate the existence of an inborn IgM mechanism deployed against a prevalent virus to reduce early post-infection viremia. In conclusion, innate IgM binding to adenovirus serotype-5 capsids restrains gene-transfer and offers a mechanism to be targeted for optimization of vector dosage in gene therapy with HDA vectors.

## Introduction

The immune system poses an important obstacle for the success of gene therapies with viral vectors [1], [2]. Conceivably, the immune system has evolved to fight prevalent microbial intruders which are often used as vehicles for therapeutic transgenes. Hepatotropic gutless or helper-dependent adenovirus serotype-5 (HDA) vectors are, in principle, ideal candidates to correct inborn metabolic diseases by reintroducing a correct version of the gene into hepatocytes [3]–[5] or by taking over the liver as a biofactory to produce a deficient plasmatic protein [6]. HDA vectors are praised for their capacity to accommodate long transgenes and for their low immunogenicity due to the absence of any viral protein from their genome with the exception in the administered packaging capsids.

Innate immunity neutralizes 90% of adenoviral particles within 24 hours following intravenous vector administration [1]. This is considered to be the result of innate up-regulation of interferons and other inflammatory cytokines via Toll-like receptor–dependent and -independent pathways [7]–[10] as well as clearance by macrophages [11]. The complement system also plays a role in adenoviral vector opsonization and clearance as a part of the innate immune system [12], [13]. IgM natural antibodies serve as a potent complement activators.

The intensity of the inflammatory innate immune response to adenoviral vectors is activated in a dose-dependent manner [1], [14]–[17]. To improve both safety and feasibility of HDA-mediated gene therapy, strategies to prevent the cytokine-mediated acute toxic response [18] or to reduce as much as possible the required viral doses are badly needed [6], [19]–[23].

Natural antibodies, predominantly IgM, provide a first line of immune defense following infection, prior to the generation of adaptive, high-affinity humoral responses. IgM is the second most abundant immunoglobulin in serum (in the range of 1–2 mg/ml) and is the dominant subtype in early humoral immune responses [24]. Since in most instances these immunoglobulins have not undergone affinity maturation through somatic hypermutation, they rely on a pentameric structure to increase valency and hence avidity for antigen [25], [26]. Thus, IgM is 100 to 10,000 times more effective than IgG in mediating agglutination. Some of the IgMs present in the serum have been produced in a T helper-independent manner and can be present even without previous exposure to cognate antigen such as those against A and B blood group antigens [27], [28]. These natural antibodies probably occur because of crossreactions with microbial flora antigens [27]. Furthermore, IgMs have been reported to bind and hamper infection by adenoviruses in an apparently antigen-independent fashion [29]. Moreover, an example of rat IgM promiscuously binding carboxyterminal ends in bacterial phages [30] has been described. IgM is able to activate the classical activation pathway of complement and thus opsonize and maybe destroy virions. An emerging picture is that IgMs may contribute to a first line of defense because they promiscuously reduce the efficiency of infection by a number of virus species before specific immunity has been developed, thus reducing viremia upon viral entry and during the first rounds of viral replication.

IgM, as well as the majority of natural antibodies, are polyreactive and bind to a number of different antigens with low affinity (Kd = 10^−4^ to 10^−7^ M). However, their low affinity to adenovirus 5 is partly compensated for by its high avidity due to pentameric structure. While the pentameric IgM demonstrated strong binding to the adenovirus capsid, monomeric IgG shows weak binding to adenovirus 5 [29]. Indeed, there is evidence for complement factor C3 activation during the elimination of adenovirus 5 by Kupffer cells [29].

It has been reported that coagulation factor X binds to the adenovirus 5 hexon via an interaction between the factor X Gla domain and the hypervariable regions of the hexon capsid protein [31]. The formation of this complex was found to be essential for transduction of the livers of mice, but factor X was not required for liver transduction in mice that lack antibodies. In a recent report [32], the ability of the first generation adenoviral serotype-5 capsids to bind factor X of coagulation has been shown to inhibit complement deposition and viral inactivation upon exposure to IgM. Non-immune IgM was found to directly bind to adenovirus 5 capsids [29]. IgM was prevented from activating complement by the clotting factor X without involvement of the protease activity of this complement factor [32]. Deficiency in factors of the classical complement activation route or complement depletion with cobra venom toxin reestablished gene transfer levels in the absence of coagulation factor X [32]. Previous reports [33]–[35] had demonstrated that in the absence of immunoglobulins, as occurs in Rag-deficient mice, there was a notorious increase in liver gene transfer by serotype-5 first generation adenovirus. A more recent work [11] showed that partial reconstitution of IgM in Rag deficient mice resulted in significant reductions in liver transduction by adenovirus 5 but not an adenoviral 5 vector engineered with the hexon from adenovirus capsid 6.

In this study we found that non-immune serum IgM is critical for reducing the efficiency of HDA vectors to mediate liver gene transfer. This is the result of an innate IgM function, which is apparently unrelated to its well described adaptive immunity functions.

## Materials and Methods

### Ethics Statement

Experimental protocols were performed according to European Council Guidelines. Acceptable standards of humane animal care and treatment employed in these mice (ref. no. CEEA069-10, CEEA068-10 and CEEA034-10) and the experimental design of this study (study number CEEA029/09 and CEEA022/13) were approved by the Ethics Committee for Animal Testing of the University of Navarra.

### Mice strains

Rag1^−/−^ B6 (B6.129S7-Rag1^tm1Mom/J^, hereafter B6.Rag1KO), Rag1^−/−^ BALB/C (C.129S7(B6)-Rag1^tm1Mom/J^, hereafter Rag1KO) and Rag2^−/−^IL-2Rγ^−/−^ BALB/C (BALB/c-Rag2^tm1Fwa^ II2rγ^tm1Wjl^, hereafter Rag2KO) transgenic mice were obtained from Jackson Laboratories (Bar Harbor, Maine USA). Female BALB/C WT and athymic (Hsd:Athymic Nude-Foxn1^nu^) mice of 6–8 weeks-old were purchased form Harlan Interfauna Iberica (Spain). µMT (B6.129S2-Ighm^tm1Cgn^/J, hereafter B6.µMT) female mice were kindly provided by Claude Leclerc (Institut Pasteur, Paris, France) and Stephanie Hughes (University of Geneva, Switzerland).

### Production of HDA vectors

The expression cassette of HDA vector contains the firefly luciferase gene under the control of the liver-specific human α-1-antitrypsin promoter with regulatory sequences from the human albumin enhancer. We also produced a second HDA vector expressing the optimized human cDNA of the housekeeping porphobilinogen deaminase (*hPBGD*) isoform directed by the same promoter. Construction and production of HDA vectors were performed as detailed elsewhere [23]. HDA-*luciferase* production had 2.15×10^12^ viral particles (vp)/ml as measured by spectrophotometry. Quantification of infective units (iu) was performed in the Huh7 cell line as detailed elsewhere [36]. The iu/vp ratio was estimated at 1/417. HDA-*hPBGD* production had 5×10^12^ vp/ml and 2.5×10^10^ infective units (iu)/ml. A dose of 5×10^10^ vp/mouse intravenously injected (iv) was used for all the *in vivo* experiments unless specified. Helper contamination was lower than 0.0001% in both productions.

### Detection of transgene expression

Non-invasive luciferase expression measurements were performed in living mice 2 days after HDA administration, as previously described [37]. Then, mice were sacrificed and DNA was extracted from liver samples using the Qiamp DNA kit (Qiagen). Liver gene transfer was analyzed by real time QPCR for detection of the 3′UTR-polyA region of the vector using specific primers (pHDAfw 5′-GCTAGCCTTTGAATGTAACCA-3′, pLUCrv 5′-ACACCTTCAGAACTGGTTTATTAGT-3′). Normalization to genomic DNA was performed using specific primers for murine GAPDH (GAPDHfw: 5′-CCAAGGTCATCCATGACAAC-3′; GAPDHrv: 5′-GCTCATTTCCTGGTATG-3′). QPCR was performed in an iQ5 real-time PCR detection system (Bio-Rad, Hercules, CA) using iQ SYBR green supermix. PCR amplification conditions were as follows: 95°C for 5 min, 40 cycles of 95°C for 15 sec, 60°C for 15 sec, 72°C for 25 s and 80°C for 10 s; and a final extension for 10 min at 72°C). The amount of each transcript was expressed according to the formula 2^Ct(GAPDH)-Ct(luciferase)^, where Ct is the cycle at which the fluorescence rises appreciably above background fluorescence.

Luciferase activity was also analyzed in liver homogenates using a luciferase assay kit (Promega) and a tube luminometer (Lumat LB 9507; Berthold Technologies, Bad Wildbad, Germany), as described elsewhere [37].

Liver expression of the PBGD transgene was measured by enzymatic activity [23] and by real-time QPCR for detection of the 3′UTR-poly region of the vector using the specific primers (pHDAfw 5′-GCTAGCCTTTGAATGTAACCA-3′; pPBGDrv 5′-CCTTCAGAACTGGTTTATTAGTAGG-3′). Normalization to genomic DNA was performed as detailed above.

### Antibodies

Polyclonal serum IgG were purchased from Sigma (ref: I5381) while polyclonal IgM were purified from BALB/c WT serum. Briefly, serum pools from 30 mice were chromatographed on a PD-10 column equilibrated with binding buffer (following GE Healthcare protocol instructions, Uppsala Sweden). Then, collected fractions with IgM were chromatographed on HiTrap IgM Column (GE Healthcare); after extensive washing IgMs were eluted according to manufacturer's instructions. The IgM batches were passed again through a PD-10 column equilibrated with PBS and eluted fractions chromatographed over protein A and protein G affinity columns. Non bound antibodies, corresponding to IgMs were collected and concentrated through 100.000 MWCO Ultracel YM-100 Centricons (Millipore, USA). Recovered IgM were finally dialyzed overnight in PBS at 4°C. The final concentration was 1.45 mg/mL as determined on a NanoDrop® ND-1000 Spectrophotometer at 280 nm with an extinction coefficient of 1.18.

Monoclonal IgG and IgM were obtained from mouse hybridomas that respectively specifically recognized human interferon alpha 5 and human AE2 anion exchanger [38] (GenBank: AAC50964.1). Hybridomas were grown in RPMI-1640 media supplemented with 10% fetal bovine serum (Ultra Low IgG; GIBCO; Cat No. 16-250-078), antibiotics, L-glutamine, Sodium Pyruvate and non-essential aminoacids. Once hybridoma cells were optimally expanded in order to eliminate bovine contaminants from the final product, the percentage of fetal bovine serum in the culture medium was progressively reduced to 5% and three days before collecting the culture supernatant, hybridoma cells were washed with phosphate buffered saline and cultured in serum-free medium. This ensures that there is no contaminant bovine IgM in the final product. Then, antibodies in the supernatant were precipitated with ammonium sulphate, resuspended and dialyzed in sodium phosphate pH 7. Immunoglobulins were then captured on Ceramic Hydroxyapatite, washed and eluted with a phosphate gradient. The eluted antibodies were passed through a protein G column. To recover the IgG, the protein G column was washed with phosphate buffered saline pH 7.4 and IgGs were eluted with Citrate Buffer pH 2.7 and neutralized with 1M Tris pH 9. For IgM recovery, the protein G column was dialyzed in 100 mM sodium acetate buffer pH 5.5. Then, collected fractions with IgM were injected into a CM Ceramic Hyper D cation exchange column (Pall Life Sciences), washed and eluted with increasing sodium chloride concentrations. Citrate buffer (IgG) or acetate buffer (IgM) were changed to saline through a PD-10 gel exclusion chromatography column, fractions pooled, and filtered through a 0.2 µm filter. Finally, immunoglobulins were dialyzed overnight in PBS at 4°C. The final monoclonal concentration was 1.57 and 1.2 mg/mL for IgM and IgG, respectively. The purity of each antibody was determined by running aliquots on a denaturing gel (SDS-PAGE) without reducing agents followed by Coomassie blue staining (figure not shown).

### B cell depletion

Antimouse-CD20 (kindly provided by Genentech, CA) was *i.v.* administered in Female BALB/C WT mice 15 and 5 days before HDA at a dose of 250 ug. To check B cell depletion, mice blood samples were stained for 10 minutes with anti-CD19-PE and FcBlock (BD Pharmingen, San Agustín de Guadalix, Spain) and erythrocytes lysed with FACS lysing solution (BD Biosciences, San Agustín de Guadalix, Spain). FACSCalibur (BD-Biosciences, San Agustín de Guadalix, Spain) was used for cell acquisition and data analysis was carried out using FlowJo software (Tree Star Inc., Olten, Switzerland).

### ELISA

Ninety-six-well plates were coated at 4°C overnight with 3×10^9^ vp of HDA-*luciferase* vector in bicarbonate buffer, pH 9.5. After washing 5 times with 2% bovine serum albumin (BSA) in PBS, plates were blocked with 2% BSA in phosphate-buffered saline for 3 hours and a half at RT. Plates were washed 10 times in washing buffer (phosphate-buffered saline +0.05% Tween20) and incubated for 2 h and a half at 4°C with 10 µg/ml of each mono- or polyclonal purified immunoglobulins (immunoglobulin added to reaction 1 µg) diluted in assay diluent (phosphate-buffered saline +2% BSA +0.05% Tween20). 10 µl of serum from WT or Rag1KO mice were diluted 1/10 in assay buffer. Plates were washed 10× in washing buffer and then incubated for 2 h at 4°C with horseradish peroxidase (HRP)-conjugated goat anti-mouse IgM (1∶4000; Pierce, Rockford, IL, USA) or anti-mouse IgG (1∶4000; Pierce, Rockford, IL, USA) antibodies at 0.2 µg/ml diluted in assay diluent. For all ELISAs, HRP was detected with tetramethylbenzidine. The same ELISA setting without HDA coating was used to detect and subtract the background signal. This technique with modifications has been previously described by R. Khare et al [11].

### *In vitro* Huh7 cells transduction

Huh7 cells were seeded in regular 6-well cell culture plates at a density of 5×10^5^ cells per well. Twenty-four hours later, 5 µg of the different immunoglobulins or 20 µl of WT serum were incubated with HDA-*luciferase* (MOI 50.000) for 30 min at 37°C with shaking. Then, Huh7 cells were treated with each Ig-vector mix and incubated all together for 4 h at 37°C in DMEM 2% heat-inactivated fetal bovine serum (Ultra Low IgG; GIBCO) in a total volume of 300 µl. Cells were then washed 3 times with saline, harvested and viral DNA was extracted using the Viral DNA extraction kit (Roche).

### Statistical analysis

Samples were run in duplicate or triplicate. The results were plotted as interquartile range (box) and extreme values (whisker). Statistical analyses in *in vivo* assays were performed using the Kruskal–Wallis non-parametric ANOVA. *Post hoc* pairwise comparisons of groups were performed using Mann-Whitney tests plus the Bonferroni correction for multiple comparisons. For the *in vitro* assays, data were log transformed prior to ANOVA analysis to equalize variances and pairwise comparisons were made using Bonferroni's Multiple Comparison Tests. The null hypothesis was rejected when P was greater than or equal to 0.05.

## Results

### A component of adaptive immunity reduces gene transfer with HDA vectors

A first generation adenovirus serotype 5 vector encoding firefly luciferase under the early CMV promoter (Vector Biolabs, Philadelphia, USA) is known to selectively transduce hepatocytes giving rise to quantifiable bioluminescence. As can be seen in Figure S1A (left panel) it was repeatedly observed that the level of gene transfer upon *i.v.* injection of 5×10^10^ vp/mouse was higher in T and B cell deficient Rag1KO mice in comparison to wild-type (WT) syngeneic mice. WT animals had not been exposed to adenovirus and were bred in Specific Pathogen-free conditions. Data in previous reports [11], [33]–[35] and those in Figure S1A (left panel) conclude that the level of gene transfer upon *i.v.* injection of a first generation adenovirus 5 vector is higher in T and B-cell deficient Rag1KO mice when compared to WT syngeneic mice. To ascertain if gene transfer was the limiting factor for lower transgene expression in WT mice, we analyzed the quantity of adenoviral DNA vector 7 days following viral transfer. Figure S1A (right panel) shows that quantitative PCR amplification of viral DNA normalized by the housekeeping gene GADPH was significantly higher in the Rag1KO mice thus pointing to less efficient gene transfer in WT mice.

**Figure 1:**
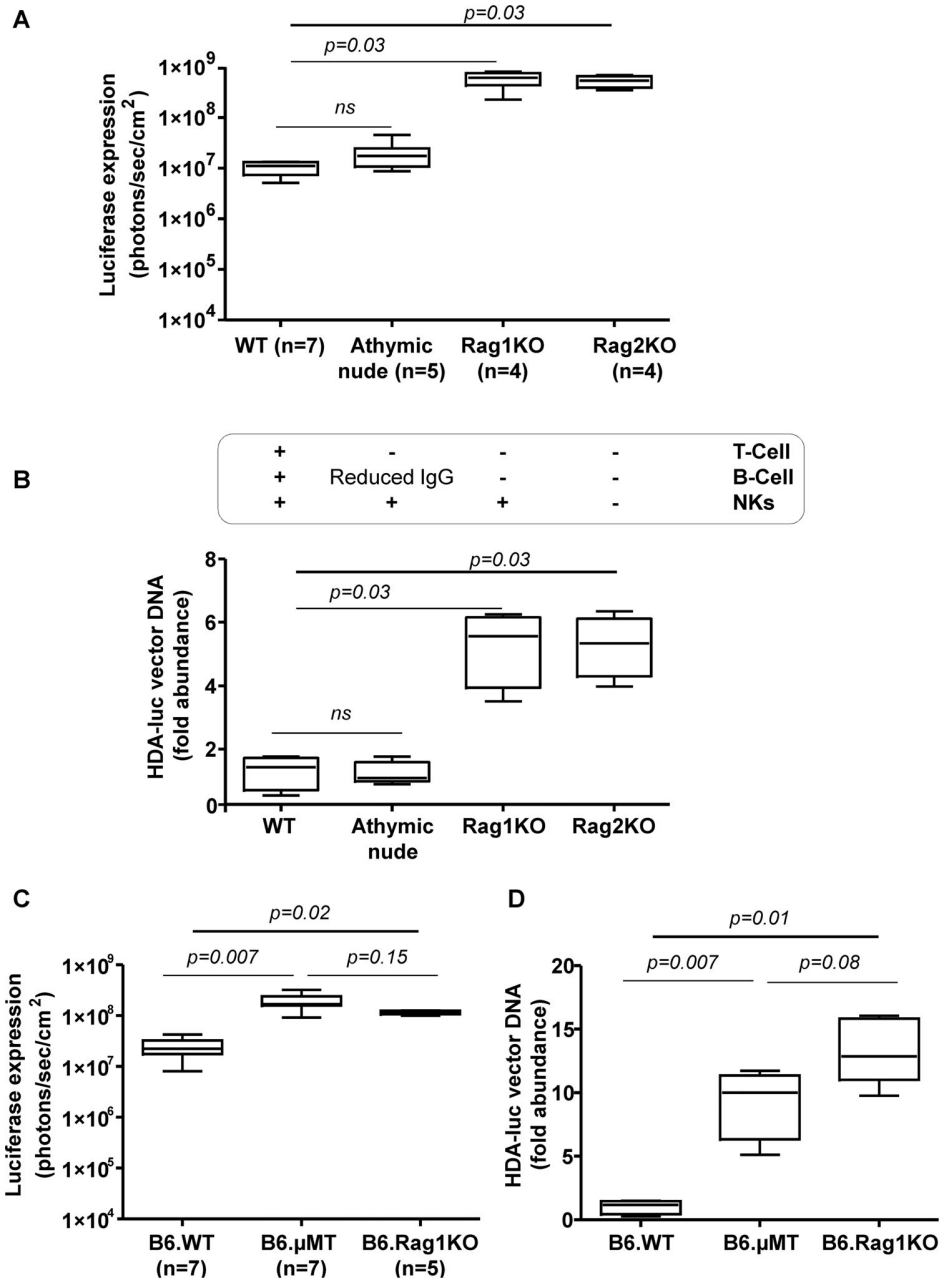
B cells are responsible for the reduction of liver gene transfer efficiency. A) Bioluminescence experiments, assessing *in vivo* luciferase activity in the liver region of mice injected *i.v.* with HDA encoding luciferase under a liver specific promoter (5×10^10^ viral particles/mouse) 48 h prior the luciferase measurement assay (similar differences were observed on days 4 and 6 -data not shown-). Mice were WT BALB/C mice or belonged to the indicated mutant strains in the background. The table under the graph indicates the immune cells absent in each modified strain. B) Relative plasmid DNA content measured by Quantitative PCR in the liver of mice shown in A excised on day 7 following vector administration. C) *In vivo* luciferase expression in the C57/BL6 mouse strain including WT, Rag1KO and µMT-KO mice that are selectively deficient in B cells. D) Experiments in mice shown in C performed to quantify viral DNA content in the liver as in B. WT, Wild-type; Rag1KO, BALB/C Rag1^−/−^ (C.129S7(B6)-Rag1^tm1Mom/J^; Rag2KO, BALB/C-Rag^−/−^IL-2Rγ^−/−^BALB/C; B6µMT, (B6.129S2-Ighm^tm1Cgn^/J; B6.Rag1KO, Rag1^−/−^ B6 (B6.129S7-Rag1^tm1Mom/J^, NKs, natural killer; IgG, immunoglobulin G; HDA-*luc*, helper-dependent adenoviral vectors encoding luciferase. Experiments were performed twice.

We analyzed if RagKO mice also fared better upon injection of an HDA encoding luciferase. For this purpose, we constructed and produced an adenovirus 5-based HDA vector encoding luciferase under liver-specific transcriptional control which encompasses the albumin enhancer and the alpha1-antitrypsin promoter (see Material and Methods). Of note, this promoter has previously been shown to be resistant to elimination or epigenetic silencing for periods of time over 450 days ( S2). Indeed, we also observed that Rag1KO mice expressed the reporter transgene more brightly (Figure 1A and Figure S1B-left panel) and incorporated viral DNA with more efficiency (Figure 1B and Figure S1B-right panel) after the *i.v*. administration of 5×10^10^ HDA-*luciferase* vp. The decrease in gene transfer was of the same order of magnitude for first and third generation adenoviral vectors that only shared the capsid proteins and suggests an innate or early adaptive function mediated by T and/or B cells.

### A function of B cells mediates gene transfer attenuation by HDA vectors

Using mice deficient in a variety of immune system cells, we next asked which functions played a role in the reduction of gene transfer by HDA in WT mice. Experiments in Rag2^−/−^IL-2γ^−/−^ mice which are deprived of NK and NKT cells as well as B and conventional T cells did not show any further increase in gene transfer after *i.v.* administration of a dose of 5×10^10^ vp/mouse (Figure 1A and B) ruling out a role for NK lymphocytes in the phenotype. The other lymphocyte candidates tested were T cells and for this purpose athymic nude mice of BALB/C background were tested in comparison to Rag1KO, Rag2^−/−^IL-2γ^−/−^ and WT mice. It was clearly observed that T-cell deficient athymic mice had similar levels of gene transfer when compared to WT mice thus excluding any involvement of conventional T lymphocytes (Figure 1A and B).

Taken together, our results indicated a key role for B cells. This was demonstrated using B-cell deficient mice which lack the µ heavy chain gene (B6.µMT) and which, accordingly, do not develop a mature B cell compartment [39]. In this mouse, we observed a phenotype clearly comparable to that of Rag1KO mice in terms of liver gene transfer by the HDA vector. It was then assumed that an antibody-production function of B cells was involved (Figure 1C and D).

### IgM from non-immune sera reduces gene transfer by HDA vectors

**Figure 2:**
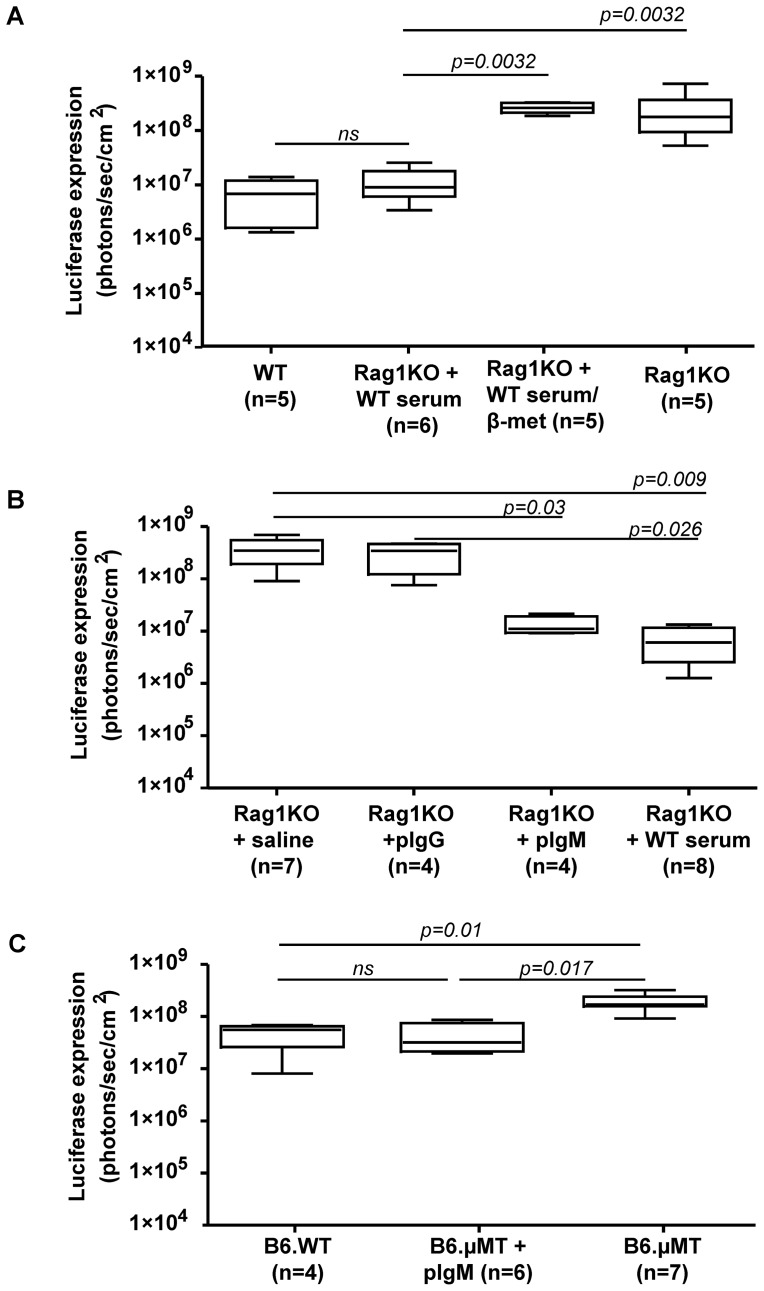
IgM mediates the mitigation of liver gene transfer by a HDA vector. A) Rag1KO mice were passively *i.v.* transferred with serum of non-immunized WT mice or similar serum samples reduced by β-mercaptoethanol. Level of gene transfer of WT mice is provided as a control. Liver gene transfer of luciferase by HDA vector (dose of 5×10^10^ viral particles/mouse) was assessed by bioluminescence as in Figure 1. B) Experiments as in A but passively transferring 2 doses of 130 μg of IgG and IgM (both purified by affinity chromatography from serum) or 400 μl of serum from WT mice. C) B6.µMT-KO mice were passively transferred *i.v.* with 2 doses of 130 μg of serum IgM purified by affinity chromatography and analyzed for liver gene transduction as in A. Wild-type, WT; Rag1KO, BALB/C Rag1^−/−^ (C.129S7(B6)-Rag1^tm1Mom/J^; B6.µMT, (B6.129S2-Ighm^tm1Cgn^/J; B6.Rag1KO, pIgG, polyclonal immunoglobulin G; pIgM, polyclonal immunoglobulin M; HDA-*luc*, helper-dependent adenoviral vectors encoding luciferase. Experiments were performed twice.

The simplest way to ascertain the involvement of antibodies was by performing an experiment involving the passive transfer of serum. For this purpose two doses of 400 μl of serum from non-immunized WT mice transferred the relative resistance to HDA liver gene transfer to Rag1KO mice of 5×10^10^ vp/mouse in a range comparable to that observed in WT mice (Figure 2A). As disulphide bridges in proteins can be disrupted by reducing agents such as β-mercaptoethanol and IgM antibodies are known to be particularly sensitive [40], we pretreated mouse sera from naïve mice with β-mercaptoethanol 0.1M at 37°C for 30 min and found that its ability to transfer resistance against HDA gene transfer was abolished (Figure 2A).

Our next question was which serum component related to B cells was responsible for our observations. Affinity purification of antibody subclasses was performed using commercially available kits and passive transfer of two doses of 130 µg of purified IgG and IgM from naïve WT mice to Rag1KO mice. The results in Figure 2B clearly show that the partial inhibition of gene transduction was transferred with the polyclonal IgM fraction but not by the polyclonal IgG fraction. Furthermore, the IgM fraction also rescued the HDA-mediated gene transfer inhibition in mice deficient in B cells as observed in knock-out animals for the μ heavy chain (Figure 2C).

Given the fact that all the animals were adenovirus naïve, our results indicate that either natural antibodies or an innate (non-adaptive) function of IgM mediates this phenomenon.

### Monoclonal IgM of known specificity distinct to adenovirus reduces HDA genome uptake in vitro

To determine if HDA gene transfer reduction by IgM was a function of natural anti-adenovirus antibodies raised in the absence of immunization or the result of an innate function of IgM, we made use of a mouse monoclonal IgM recognizing human AE2 anion exchanger obtained from a conventional clonal hybridoma (see methods) free of bovine IgM and totally unrelated to adenovirus capsid antigens.

**Figure 3:**
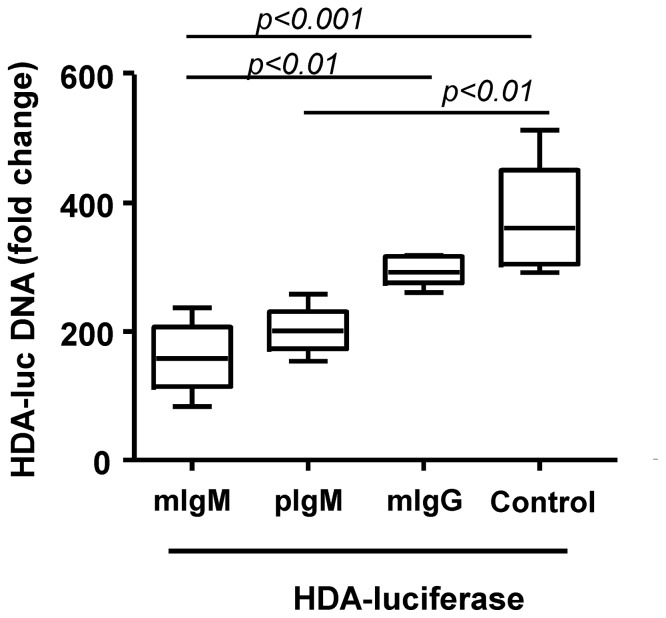
Monoclonal IgM inhibits gene transfer of Huh7 hepatocellular carcinoma cells. *In vitro* gene transfer of 2.5×10^10^ vp of HDA-*luciferase* to Huh7 cells (MOI 50.000) in the presence of 2% fetal bovine serum and 5 µg of monoclonal IgM (mIgM) directed to an irrelevant antigen (non crossreactive human AE2 transporter), monoclonal IgG which specifically recognized human interferon alpha-5 or serum polyclonal IgM from adenovirus naïve mice (pIgM) as indicated. Relative content of vector DNA was assessed by real time PCR in quadruplicate samples. pIgM, polyclonal immunoglobulin M; mIgM, monoclonal immunoglobulin M, mIgG, monoclonal immunoglobulin G; HDA-*luc*, helper-dependent adenoviral vectors encoding luciferase. The experiment was repeated three times.

**Figure 4:**
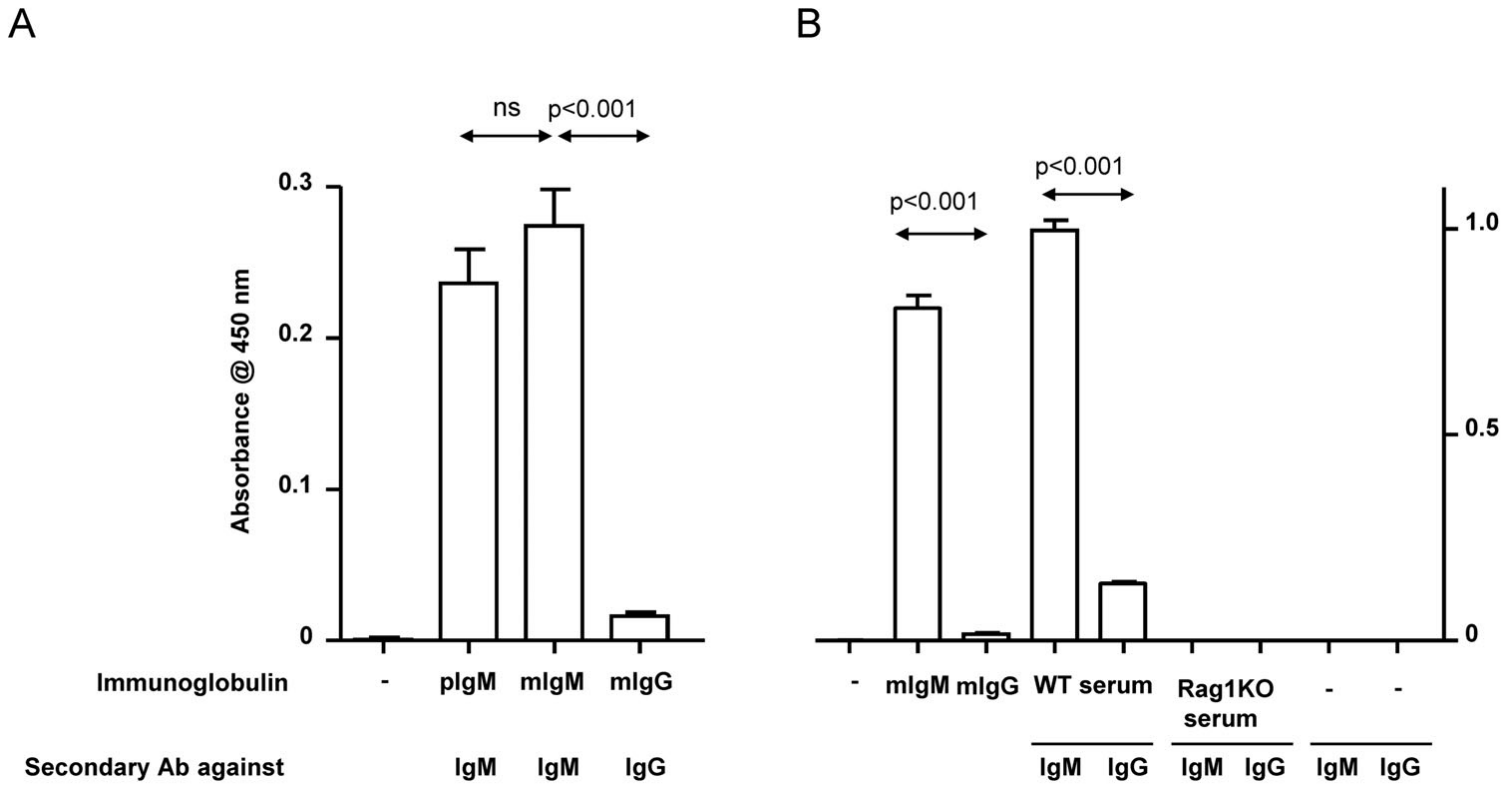
IgM antibodies bind adenoviral capsids. ELISA tests performed on HDA-*luciferase* coated 96-well plates sequentially incubated with the indicated antibodies and secondary antibodies. Results are presented as absorbance at 450 nm ± standard deviation at the time of stopping the reaction. A) Binding of the indicated polyclonal non-immune IgM and the monoclonal hybridoma-produced IgM and IgG were tested for their ability to bind capsids and were revealed with the proper subclass specific secondary reagents. B) ELISA Experiments as in A extending the developing time to detect potentially weaker interactions and testing WT and Ig-free Rag1KO serum as well as monoclonal IgG and IgM. The background reactivity of secondary antibodies was virtually non-existent. Wild-type, WT; Rag1KO, BALB/C Rag1^−/−^ (C.129S7(B6)-Rag1^tm1Mom/J^; pIgM, polyclonal immunoglobulin M; mIgM, monoclonal immunoglobulin M, mIgG, monoclonal immunoglobulin G. Experiments were repeated twice in replicates and statistical comparisons were performed as described in Materials and Methods section.

It was found that both polyclonal IgM from serum and monoclonal IgM from the anti-AE2 hybridoma inhibited *in vitro* gene transfer of the Huh7 hepatocellular carcinoma cell line mediated by the luciferase encoding HDA (2.5×10^10^vp/condition). A decrease in the HDA genome uptake was also observed when IgM was compared with monoclonal IgG- in this *in vitro* assay, although statistical significance was only reached with monoclonal IgM (Figure 3). In order to provide a molecular explanation, we performed an ELISA (Figure 4A and B) to determine if HDA capsids adsorb IgM antibodies. It was observed that both monoclonal and polyclonal IgM bind adenoviral capsids, whereas monoclonal IgG showed almost no binding to HDA (Figure 4A). Non-immune serum showed similar levels of polyclonal IgM binding to HDAs capsids in the ELISA assays (Figure 4B).

Another corollary of the critical involvement of B cells is that we could test if depletion of this subset in adult mice would rescue more efficient gene transfer (Figure S3A). For this purpose, we performed extensive depletions of CD20 lymphocytes (Figure S3B) with a specific monoclonal antibody. Depletion did not reduce IgG serum concentrations, while IgM was only decreased to about one half of the original concentration (Figure S3C and D). However, almost complete B cell depletion under these conditions was not able to rescue gene transfer, implying that the remaining IgM concentration was sufficient to inhibit gene transfer.

### Monoclonal unspecific IgM reduces in vivo gene transfer with HDA

**Figure 5:**
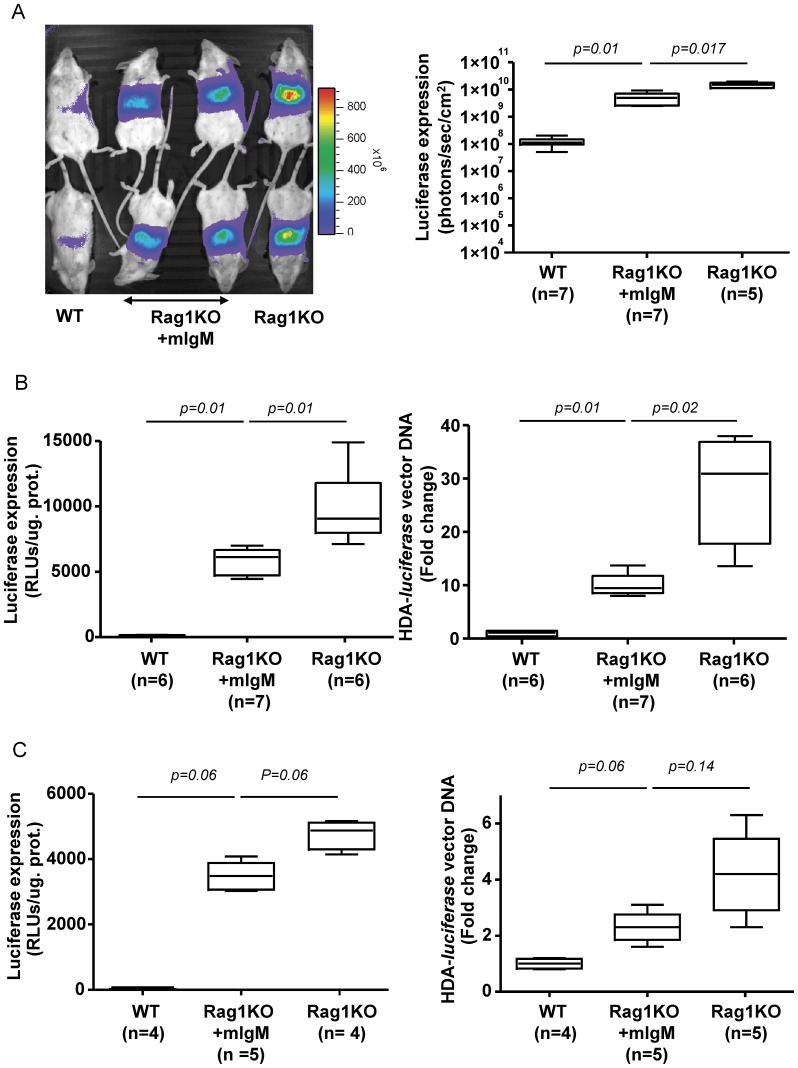
Monoclonal IgM of a specificity unrelated to adenovirus reduces liver gene transfer by HDA vectors. A) Representative photographs of light emission from the liver region of mice intravenously injected with 5×10^10^ vp/mouse 48 h prior to bioluminescence (left panel). When indicated, Rag1KO were pre-injected twice with 130 µg of monoclonal IgM of irrelevant specificity. The dot graph on the right shows quantitative data from the same experiment. Experiments B and C were performed seven days following gene transfer with progressively reduced doses of HDA-*luciferase* (3.6×10^10^ vp/mouse and 2.2×10^9^ vp/mouse, respectively). Gene transfer was assessed by measuring luciferase activity ex-vivo in homogenates from liver samples (left panel). The relative liver content of vector DNA was measured by quantitative PCR (right panel). WT, Wild-type; Rag1KO, BALB/C Rag1^−/−^ (C.129S7(B6)-Rag1^tm1Mom/J^; pIgM, polyclonal immunoglobulin M; mIgM, monoclonal immunoglobulin M, mIgG, monoclonal immunoglobulin G.; HDA-*luciferase*, helper-dependent adenoviral vectors encoding luciferase; RLU, Relative Light Unit. Experiments were performed twice.

We next tested if the monoclonal IgM antibody of unrelated specificity also mediated gene transfer inhibition *in vivo,* when passively transferred to immunoglobulin deficient Rag1KO mice. Two doses of 130 μg of purified IgM antibody were administered 24 h and 2 h before 5×10^10^ vp of HDA-*luciferase*. The injection of monoclonal IgM curtailed HDA gene transfer in terms of luciferase activity and DNA content in the liver (Figure 5). This inhibitory effect of monoclonal IgM was more prominent when a relatively high *i.v.* dose of HDA was used (Figure 5B and C). Luciferase expression in the liver was reduced by 42% and 27% in mice *i.v.* injected with 3.6×10^10^ vp/mouse and 2.2×10^10^ vp/mouse of HDA-*luciferase*, respectively. Furthermore, vector DNA content was reduced by 55% or 36% in the liver of IgM treated-Rag1KO mice injected with either high or low doses of the HDA vector as measured seven days after vector administration. These data suggested that the deleterious effect of IgM is more prominent with relatively high therapeutic doses HDA.

**Figure 6:**
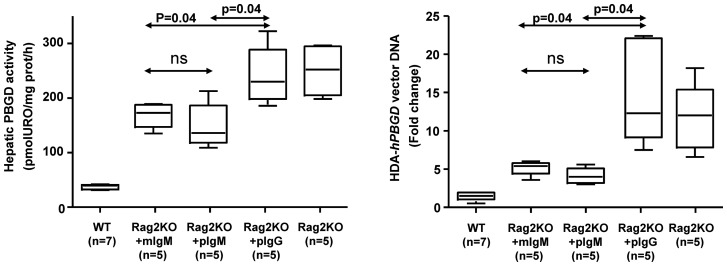
*In vivo* gene transfer of a HDA encoding PBGD is inhibited by monoclonal and polyclonal IgM. Gene transfer with a HDA vector intended for the correction of acute intermittent porphyria were performed in Rag2KO mice or control WT mice. Immunoglobulins were injected 24 h and 2 h before the infusion of 5×10^10^ vp/mouse of HDA-PBGD. PBGD enzymatic activity in liver homogenates obtained 48 h after gene transfer (left panel) and PBGD transgene relative DNA content to GADPH by quantitative PCR (right panel). WT, Wild-type; Rag2KO, Rag^−/−^IL-2Rγ^−/−^; pIgM, polyclonal immunoglobulin M; mIgM, monoclonal immunoglobulin M, mIgG, monoclonal immunoglobulin G; HDA-*PBGD*, helper-dependent adenoviral vectors encoding human porphobilinogen deaminase.

Finally, the polyclonal IgM activity to interfere with HDA gene transfer was compared to that of monoclonal IgM and IgG in Rag2KO mice (Figure 6). For this purpose, mice were injected with a HDA carrying the human porphobilinogen deaminase gene using the same promoter used in HDA-*luciferase* [23]. This vector is more relevant as it is intended for treatment of acute intermittent porphyria. Results in figure 6 support that passive transfer of both monoclonal IgM of irrelevant specificity and non-immune polyclonal IgM strongly restrain gene transfer to the liver of Rag2KO mice in terms of enzyme activity in liver tissue homogenates (Figure 6, left panel) and transgene DNA content (Figure 6, right panel). In conclusion, an innate and antigen-unspecific function of IgM limits the efficiency of gene transfer by HDA vectors including those intended for liver gene transfer in clinical trials.

## Discussion

Optimization of gene transfer to the liver by recombinant gutless adenovirus is perceived as an opportunity for gene therapy for a broad spectrum of diseases [3]–[6], [41], [42]. Given the potential inflammatory complications of high doses of viral particles [15], [9], [10], [17], [43] drastically decreasing dose requirements [1], [2], [6], [19], [21]–[23] is an important objective in successful clinical translation. The innate response to adenoviral capsid is clearly complex and multifactorial [7], [8], [10]. In pre-immunized animals, antibodies binding to adenovirus contribute to the acute toxicity by opsonizing adenoviral particles and rendering them more susceptible to Fc-mediated uptake and clearance by liver macrophages [12], [13], [29].

In this study we sought to examine one such limiting factor, namely the ability of normal serum from non-immunized animals to prevent adenoviral vector infection and liver gene transfer. A methodical quest was initiated that concluded that B lymphocytes were necessary and that an immunoglobulin component was involved. Fractionation of serum experiments conclusively showed that IgM was to blame for the unwanted inhibitory effect. Importantly a monoclonal IgM specific for a human ion channel and totally unrelated to adenovirus capsids also produced this partial inhibition of gene transfer. Therefore we attribute this limiting factor to an inherent non-adaptive protein function of IgM. Results from previous reports [11], [29], [32] also show that serum polyclonal IgM binds adenovirus 5 capsids and hampers gene transfer. Our study demonstrates that there is no involvement of antigen specificity but an effect related to an innate function of IgM, also present in a monoclonal IgM that is not specific for adenovirus. Such an interpretation is further supported by the fact that another mouse monoclonal IgM directed to an unrelated glycosil-phosphatidylinositol-related antigen also binds to adenoviral capsids in ELISA assays and reduces liver gene transfer *in vivo* (data not shown). These findings make the interpretation of antibody cross-reactivity very unlikely, although we cannot definitively exclude it. The IgM region responsible for this activity remains to be mapped.

IgM is present in high concentrations in blood. It has a relatively short half-life in the serum, approximately 28 hours, in normal mice in the absence of antigen [24]. It makes evolutionary sense that IgM would host biological properties that would deal with early infection [24], [26] following virus inoculation or the first rounds of viral replication.

Little is known about the exact mechanisms accounting for our observations of gene transfer reduction. We have shown adsorption of adenovirus 5 capsids to serum IgM or monoclonal IgM but subsequent molecular events leading to gene transfer inhibition remain to be elucidated. IgM can fix and activate complement through the classical route of activation and create C3 fragments that would opsonize particles for their intake by phagocytes including liver macrophages [11], [30]. As the most abundant capsid protein, most neutralizing antibodies are directed to hexon protein. Antibodies also appear to neutralize the adenovirus not by blocking viral entry (antibodies against fiber) but instead by blocking microtubule transport of the virus to the nucleus after it escapes from endosomes (hexon-targeted antibodies) [44]. Evidence have been published showing that complement limits gene transfer by an adenovirus 5-based first generation adenoviral vector and that role is played by IgM from non-immunized mice in this effect [11], [29], [32]. Factor X of coagulation has been shown to bind adenovirus 5 capsid and protect it from attack by the classical complement pathway. As yet we have not addressed complement involvement but we have extended these observations of IgM-mediated inhibition of first generation adenoviral vectors to HDA vectors.

It must be considered that both our observations and those by Xu et al. [32] have been made with serotype 5 adenoviral vectors and it remains to be seen if this is a general property applicable to other serotypes. Khare et al [11] showed that engineered adenovirus 5 bearing the hexon from adenovirus serotype 6 may bind IgM or other immunoglobulins but evades its clearance by Kupffer cells. Mapping such an activity to subregions in the IgM constant region would be of great interest, but it is a difficult endeavor due to the fact that it is likely that both adsorption to adenovirus and complement activation would be required in the same moiety that needs to remain properly structured in heavy and light chains. We have observed that disruption of IgM with disulphide-bridge reducing agents inhibits its ability to reduce adenoviral gene transfer. We are currently exploring if non-specific multimeric IgM, the predominant form in plasma, can physically aggregate adenoviral capsids and thereby conceivably inhibit gene transfer.

Our early attempts to improve transfer by B-cell depletion with anti-CD20 mAbs failed because of the half-life of IgM and its production by CD20-negative plasma cells [45]. Indeed, attaining improvement with these approaches will be very difficult because polyclonal IgM quantities much lower than the total plasma content in WT mice (approximately 10–15%) greatly rescue the inhibition in Rag-deficient mice. In this sense 400 microliters of WT serum (approximately one fourth of the total serum in the mouse) transferred to immunodeficient mice hampered gene transfer to the level observed in B-cell immunocompetent mice.

Other strategies to reduce IgM levels prior to gene-therapy treatment in humans should be suitable. These include plasmapheresis [46] that would reduce both IgM and complement factors. This procedure is difficult to test in rodents. If the protective role of factor X is critical [32], providing an additional supply even directly into the suspension of HDA vector prior to injection may be an interesting option. Our *in vitro* gene transfer experiments to Huh7 cells were performed in the presence of 2% complement-inactivated bovine serum and under such conditions monoclonal IgM decrease gene transfer by the HDA vector. Probably in this setting IgM antagonizes interactions with viral receptors. This *in vitro* assay provides a simple model to study the molecular requirements for complement and coagulation factors and to devise strategies to counteract the innate inhibitory role of IgM antibodies for efficient gene transfer by HDA.

As a whole, our data show that IgM has innate immunity functions. These biological properties confer sufficient avidity to a wide range of viruses and viral vectors, thus constituting a mitigating factor for viral infection but also a perhaps a removable hurdle for gene transfer. In particular, IgM is an important obstacle for gene therapy with HDA vectors.

## Supporting Information

Figure S1 **The adaptive immune system decreases liver gene transfer efficiency by recombinant adenoviral vectors.** A) The indicated WT numbered strain of mice were intravenously injected with 4×10^9^ plaque-forming units of AdCMV-*luciferase*/kg and monitored 24 h later for luciferase expression in the liver region (left panel) and vector DNA content by quantitative PCR (right panel). AdCMV-*luciferase* is an E1, E3-deleted replication-deficient adenoviral vector expressing firefly luciferase under the control of the cytomegalovirus promoter (Vector Biolabs, Philadelphia, PA). B) Similar assays as in A but using a third generation helper-dependent adenoviral vector expressing luciferase under a liver-specific promoter (5×10^10^ viral particles of HDA-*luciferase*/mouse). The non-parametric Mann–Whitney U-test was used for comparison of two groups (GraphPad Prism software, Graphpad Software Inc., La Jolla, CA, USA). The results were presented as the interquartile range (box) and the extreme values (whisker). The null hypothesis was rejected when P was greater than or equal to 0.05. WT, wild type; Rag1KO, Rag1^−/−^ BALB/C (C.129S7(B6)-Rag1^tm1Mom/J^; Ad-Luc, first generation adenoviral vector expressing firefly luciferase; HDA-*luciferase*, helper-dependent adenoviral 5 vectors encoding luciferase.
(TIF)

Figure S2 **Duration of liver gene transfer in a cohort of 5 wild-type BALB/c mice injected intravenously with 5×10^10^ viral particles/mouse of HDA-** ***luciferase*** **.** Luciferase expression was periodically monitored up to 450 days using a Xenogen IVIS bioluminescence imaging system (Xenogen, Alameda, CA) which includes a cooled charge-coupled device (CCD) camera. HDA-*luciferase*, helper-dependent adenoviral 5 vectors encoding luciferase. The results were expressed as the mean ± standard deviation.
(TIF)

Figure S3 **B-cell depletion with anti-CD20 mAb does not increase gene transfer most probably due to small reductions in serum IgM concentration.** B cell depletion for 3 weeks with anti-CD20 mAb (mouse analogue to rituximab) did not increase HDA-mediated *luciferase* liver gene transfer in female BALB/C wild type mice (A) in spite of extensive reductions in B cells in the spleen (B). FACSCalibur (BD-Biosciences, San Agustín de Guadalix, Spain) was used for cell acquisition and data analysis was carried out using FlowJo software (Tree Star Inc., Olten, Switzerland). Follow up of immunoglobulin concentrations showed only 30–40% decreases in serum concentrations of IgM (C) without changes in serum IgG (D). The results were presented as the interquartile range (box) and the extreme values (whisker). Data were log transformed prior to one-way ANOVA analysis to equalize variances and pairwise comparisons were made using Bonferroni's Multiple Comparison Tests. The null hypothesis was rejected when P was greater than or equal to 0.05. WT, wild type; Rag1KO, Rag1^−/−^ BALB/C (C.129S7(B6)-Rag1^tm1Mom/J^); anti-CD20, Antimouse-CD20 antibody; HDA-*luciferase*, helper-dependent adenoviral 5 vectors encoding luciferase.
(TIF)
