# Supplementary figures and images for: Innate Functions of Immunoglobulin M Lessen Liver Gene Transfer with Helper-Dependent Adenovirus
Source: PLoS One. 2014 Jan 21;9(1):e85432. doi: 10.1371/journal.pone.0085432 (PMC3897443; doi:10.1371/journal.pone.0085432)

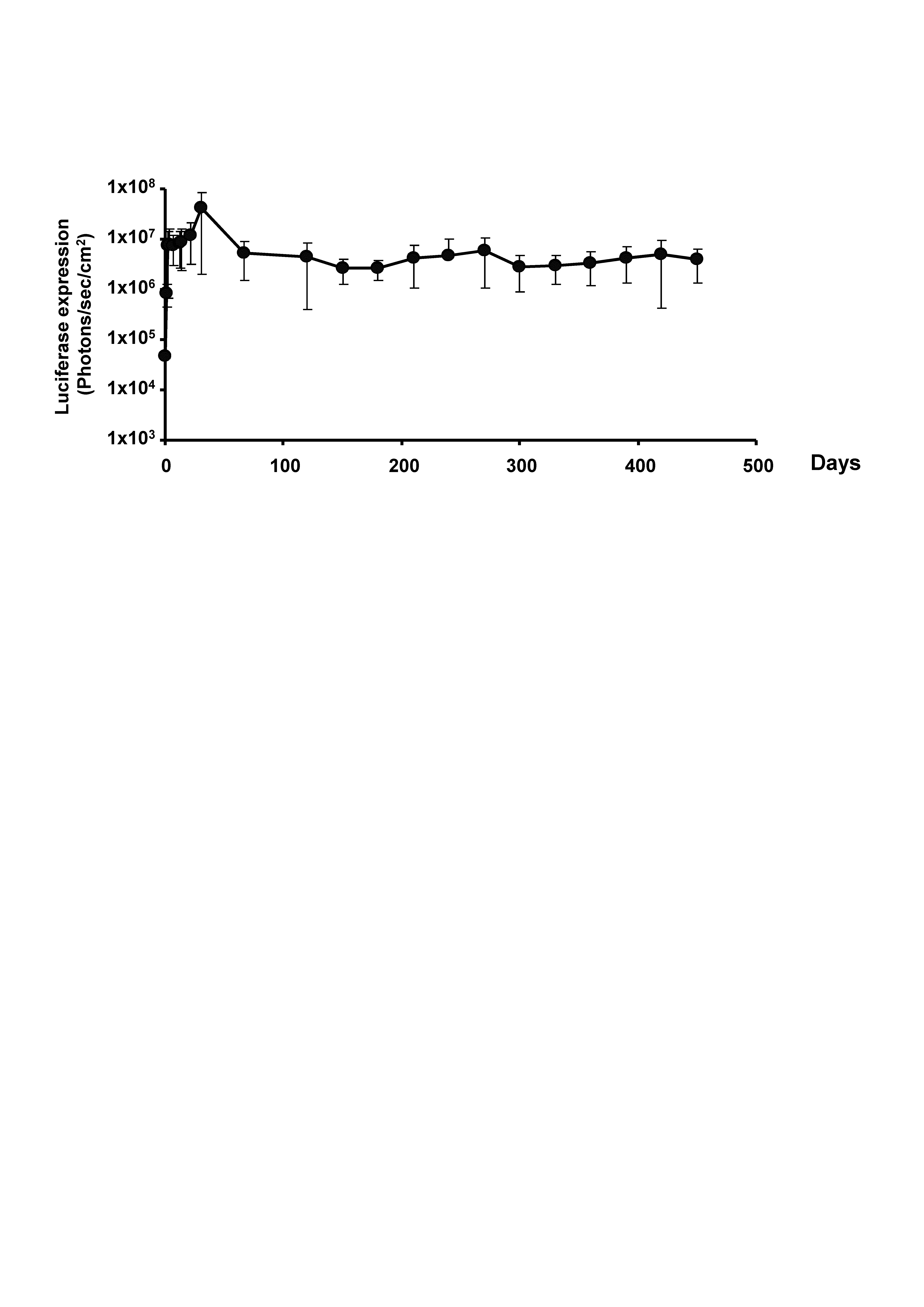

Supplement: Figure S2 — Duration of liver gene transfer in a cohort of 5 wild-type BALB/c mice injected intravenously with 5×10^10^ viral particles/mouse of HDA- luciferase . Luciferase expression was periodically monitored up to 450 days using a Xenogen IVIS bioluminescence imaging system (Xenogen, Alameda, CA) which includes a cooled charge-coupled device (CCD) camera. HDA-luciferase, helper-dependent adenoviral 5 vectors encoding luciferase. The results were expressed as the mean ± standard deviation. (TIF) [file pone.0085432.s002.tif]

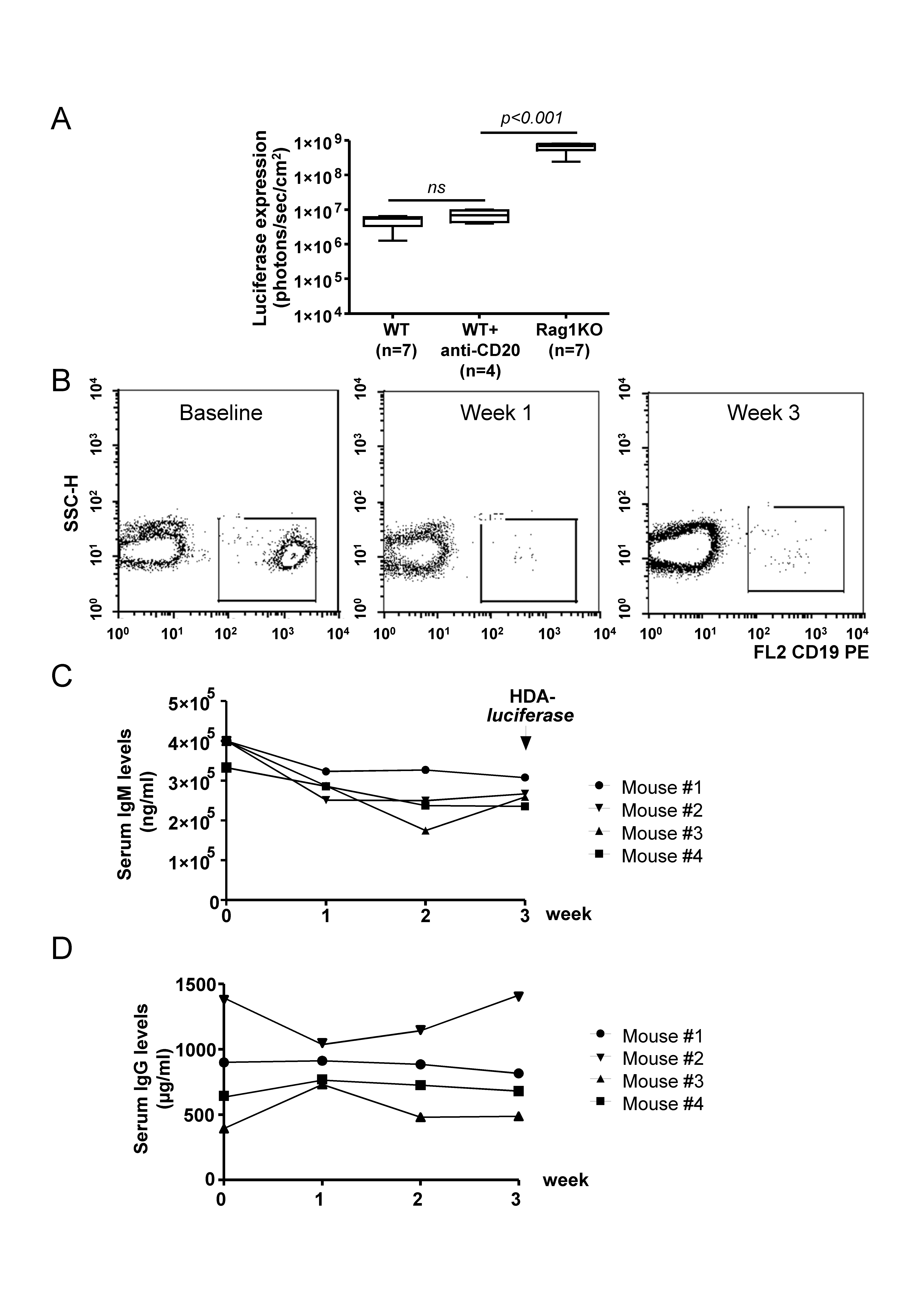

Supplement: Figure S3 — B-cell depletion with anti-CD20 mAb does not increase gene transfer most probably due to small reductions in serum IgM concentration. B cell depletion for 3 weeks with anti-CD20 mAb (mouse analogue to rituximab) did not increase HDA-mediated luciferase liver gene transfer in female BALB/C wild type mice (A) in spite of extensive reductions in B cells in the spleen (B). FACSCalibur (BD-Biosciences, San Agustín de Guadalix, Spain) was used for cell acquisition and data analysis was carried out using FlowJo software (Tree Star Inc., Olten, Switzerland). Follow up of immunoglobulin concentrations showed only 30–40% decreases in serum concentrations of IgM (C) without changes in serum IgG (D). The results were presented as the interquartile range (box) and the extreme values (whisker). Data were log transformed prior to one-way ANOVA analysis to equalize variances and pairwise comparisons were made using Bonferroni's Multiple Comparison Tests. The null hypothesis was rejected when P was greater than or equal to 0.05. WT, wild type; Rag1KO, Rag1−/− BALB/C (C.129S7(B6)-Rag1tm1Mom/J); anti-CD20, Antimouse-CD20 antibody; HDA-luciferase, helper-dependent adenoviral 5 vectors encoding luciferase. (TIF) [file pone.0085432.s003.tif]
